# Chitosan hydrogel encapsulated with LL-37 peptide promotes deep tissue injury healing in a mouse model

**DOI:** 10.1186/s40779-020-00249-5

**Published:** 2020-04-22

**Authors:** Xu Yang, Jing-Lin Guo, Jing Han, Rui-Juan Si, Pan-Pan Liu, Zi-Rui Zhang, Ai-Min Wang, Ju Zhang

**Affiliations:** School of Nursing, Qingdao University, Qingdao, 266021 China

**Keywords:** LL-37, Chitosan hydrogel, Deep tissue injury, Pressure ulcer, Cathelicidin

## Abstract

**Background:**

LL-37 peptide is a member of the human cathelicidin family, and has been shown to promote the healing of pressure ulcers. However, the low stability of this peptide within the wound environment limits its clinical use. Chitosan (CS) hydrogel is commonly used as a base material for wound dressing material.

**Methods:**

CS hydrogel (2.5% w/v) was encapsulated with LL-37. Cytotoxicity of the product was examined in cultured NIH3T3 fibroblasts. Effects on immune response was examined by measuring tumor necrosis factor-α (TNF-α) release from RAW 264.7 macrophages upon exposure to lipopolysaccharides. Antibacterial activity was assessed using *Staphylococcus aureus*. Potential effect on pressure ulcers was examined using a mouse model. Briefly, adult male C57BL/6 mice were subjected to skin pressure using magnets under a 12/12 h schedule for 21 days. Mice were randomized to receive naked LL-37 (20 μg), chitosan gel containing 20-μg LL-37 (LL-37/CS hydrogel) or hydrogel alone under the ulcer bed (*n* = 6). A group of mice receiving no intervention was also included as a control.

**Results:**

LL-37/CS hydrogel did not affect NIH3T3 cell viability. At a concentration of 1–5 μg/ml, LL-37/CS inhibited TNF-α release from macrophage. At 5 μg/ml, LL-37/CS inhibited the growth of *Staphylococcus aureus.* The area of the pressure ulcers was significantly lower in mice receiving LL-37/CS hydrogel in comparison to all other 3 groups on days 11 (84.24% ± 0.25%), 13 (56.22% ± 3.91%) and 15 (48.12% ± 0.28%). Histological examination on days 15 and 21 showed increased epithelial thickness and density of newly-formed capillary with naked LL-37 and more so with LL-37/CS. The expression of key macromolecules in the process of angiogenesis (i.e., hypoxia inducible factor-1α (HIF-1α) and vascular endothelial growth factor-A (VEGF-A)) in wound tissue was increased at both the mRNA and protein levels.

**Conclusion:**

Chitosan hydrogel encapsulated with LL-37 is biocompatible and could promote the healing of pressure ulcers.

## Background

Pressure injuries, also known as pressure ulcers, are common in bed-ridden patients, and associated with poor quality of life as well as high medical care costs [1, 2]. Pressure injuries have also been associated with poor prognosis and higher mortality in some patients [3]. Deep tissue injuries can rapidly develop into open ulcers, exposing wounded tissue to the external environment. Therefore, in clinical wound care and nursing specialties, the development of effective treatments for deep tissue pressure injuries is urgently required.

Inflammatory responses and reductions in capillary density caused by ischemia/reperfusion are the main factors that affect deep tissue injury healing [4, 5]. The main principles of deep tissue injury management are in the inhibition of inflammatory responses in conjunction with the restoration of blood microcirculation within the wound site [4, 6–8]. However, there are limited therapeutic drugs that can achieve these desirable effects; hence, there is an urgent requirement for the development of new, effective means to satisfy the unmet clinical need for the treatment of deep tissue injuries.

LL-37 is the only antimicrobial peptide of the cathelicidin family that has been identified in humans [9]. Recent studies have highlighted the important roles that antimicrobial peptides play in the regulation of wound healing [10]. In addition to bactericidal actions, LL-37 can also bind to Toll-like receptors (TLRs), inhibit TLR signaling pathways and reduce the production of proinflammatory cytokines [11]. LL-37 also contributes to blood vessel formation and has been shown to act as a practical immune adjuvant [12, 13]. Local injection of LL-37 significantly increased ischemic hind limb collateral circulation in animal models [14]. In clinical trials, it was found that supplementation with LL-37 was safe and was well tolerated when applied to nonhealing venous leg ulcers (VLUs) [15]. The major challenge associated with LL-37 administration is its rapid degradation within the wound environment; thus, treatments require higher dosage and dosing frequencies to achieve the desired therapeutic effect [16].

Recent research has shown that hydrogel dressings are an attractive option for use as small molecule drug delivery systems [17, 18]. Hydrogel dressings not only provide a moist healing environment for in vivo local injury tissues but also function as an extracellular matrix-like scaffold for cellular support while maintaining the biological activity of loaded small molecule polypeptides [19–21]. Multiple reports have shown that Chitosan (CS) hydrogels have strong potential for use in both pharmaceutical and medical applications [22, 23]. CS hydrogels are also widely used as drug delivery systems and wound dressings due to their mucoadhesive properties [24]. Previously, CS hydrogel wound dressings were prepared and successfully loaded with small molecular peptides for application in the treatment of lower limb ischemic disease [25]. In light of these reports, CS was selected as an appropriate base material for the hydrogels used in this study. Hydrogels were fabricated at a concentration of 2.5% CS (w/v), as it was previously reported that CS hydrogels at this concentration have suitable biocompatibility and fast degradation profiles [26].

In the present study, LL-37/CS hydrogel dressings were prepared using physical blending methods. The therapeutic effects were assessed, and molecular mechanisms of action were evaluated in deep tissue injuries through the use of molecular biology techniques. The results obtained in this study provide a theoretical basis for further investigations into the clinical application of LL-37/CS hydrogel dressings.

## Materials and methods

### Reagents

All reagents were purchased from Sigma-Aldrich unless otherwise stated. LL-37 (95% purity, LLGDFFRKSKEKIGKEFKRIVQRIKDFLRNLVPRTES) was synthesized by GL Biochem (Shanghai, China). NIH3T3 cells were purchased from the Chinese Academy of Medical Sciences (Tianjin, China). Fetal bovine serum (FBS) and trypsin were purchased from Gibco (Thermo Fisher Scientific, Waltham, MA, US); Medium 1640 was purchased from HyClone (GE Healthcare, Chicago, IL, US); Cell Counting Kit-8 (CCK-8) was purchased from Dojindo (Kumamoto, Japan).

### Preparation of LL-37/CS hydrogel

CS hydrogels were prepared as previously described [27, 28]. Differing qualities of LL-37 powder were gradually dissolved in phosphate-buffered saline (PBS). The CS hydrogel solution was filtered through 0.22-μm filter membranes (Millex, Merck Millipore, Burlington, MA, US), and the LL-37 solution was slowly added under magnetic stirring (600 r/min × 5 min) at 4 °C.

### Encapsulation efficiency

The encapsulation efficiency of LL-37 within CS hydrogels was determined by measuring the amount of non-encapsulated LL-37 still present within the supernatant after hydrogel formation and after 1 mg of lyophilized LL-37/CS hydrogel was dissolved in 1 ml of water. The concentration of LL-37 that was encapsulated within CS hydrogels was analyzed by HPLC using conditions reported in the literature [29]. A calibration curve was plotted for the LL-37 peptide concentration range from 1 ng/ml to 25,000 ng/ml. The LL-37 concentration was quantified, and the encapsulation efficiency of LL-37 was calculated using the following equation: Encapsulation efficiency = [Encapsulated LL-37/Initial LL-37] × 100%.

### Antimicrobial activity

Antimicrobial activity was compared among LL-37 (5 μg/ml), LL-37/CS hydrogel (LL-37, 5 μg/ml) and CS hydrogel using *Staphylococcus aureus* bacterial culture. Briefly, a total of 1 × 10^5^*Staphylococcus aureus* bacterial cells were added to 1 ml of PBS containing LL-37 or hydrogels and incubated for 6 h at 37 °C. A series of aliquots (100 μl) were taken and diluted in PBS to yield 1 × 10^3^ bacteria per ml solution, and then plated on LB agar and incubated for 24 h at 37 °C before colonies were counted.

### Cell culture

NIH3T3 cells were cultured in DMEM (Gibco) supplemented with 10% FBS containing antibiotics (100 U/ml penicillin and 100 μg/ml streptomycin. Cells were maintained at 37 °C in a humidified 5% CO_2_ atmosphere. Cells were subcultured every 2–3 days or when a confluent monolayer had formed.

### Cytotoxicity assay

Cytotoxicity to NIH3T3 cells was examined using CCK-8 assays. The experiment was grouped into: control (saline), LL-37/CS (containing various concentration of LL-37: 10 ng/ml, 50 ng/mL, 100 ng/ml), CS with 6 replicate wells per group. Briefly, cells were seeded in 96-well plates at a density of 1 × 10^5^ cells/ml, grown to 70–80% confluence, and incubated with LL-37/CS hydrogel or free LL-37 at various concentrations for 24 or 48 h. After two washes with PBS, cell viability was assessed using a CCK-8 assay kit (Dojindo). Absorbance was measured at 450 nm. The results are expressed as the percentage of control cultures.

### Enzyme-linked immunosorbent assay (ELISA)

ELISA was used to assess the release of tumor necrosis factor-α (TNF-α) from macrophages. RAW 264.7 cells (1 × 10^5^) were seeded into 96-well culture plate. After 24-h culture, the DMEM medium was replaced by DMEM containing 20 ng/ml lipopolysaccharide (LPS) and either LL-37 (5 μg/ml), LL-37 (5 μg/ml)/CS hydrogel, or CS hydrogels at an equivalent CS concentration. For negative control, 1% FBS-supplemented DMEM was used. For positive control, 1% FBS-supplemented DMEM containing 20 ng/ml LPS was used. Following an 18-h incubation time, the supernatant was collected, and TNF-α release was quantified by ELISA kit (No. 1217202, Dekewe Biotech, China).

### In vivo wound healing study

#### Animals

Male C57BL/6 mice (6–8 weeks of age, approximately 20 g) were acquired from Beijing Vital River Laboratory Animal Technology (Beijing, China), and maintained in the Qingdao University Veterinary Service Center (Qingdao, China). Experiments were performed in compliance with the guidelines established by the Institutional Animal Care and Use Committee of Qingdao University (Qingdao, China).

#### Deep tissue injury

Hair of the right hind limb of the mouse was shaved using hair clippers. An area close to the gluteus superficialis muscle was subjected to pressure using a magnet (12-mm diameter, 5-mm thickness, 2.4 g weight, 1000 G surface magnetic flux) under a 12/ h under pressure and 12 h schedule. During the 12-h period with pressure, the mice had unlimited access to food and water, and allowed to move in the cage freely. One day after the paradigm started, mice randomly received subcutaneous injection of 20-μg LL-37, 20-μg LL-37/CS, or CS hydrogel alone (*n* = 6) into deep tissue under the magnet. A group of mice received magnet but no other intervention was included as a control. On day 21, the mice were sacrificed by severing the neck to obtain tissue for further analysis.

#### Evaluation of wound healing

Wound healing was evaluated by measuring the wound area on days 1, 3, 5, 7, 9, 11, 13, 15, 17, 19 and 21. Wound sites were photographed, and the wound area was measured using ImageJ analysis software (NIH, Bethesda, MD, US). Wound closure was expressed as the percentage of the initial wound area. The healing ratio was calculated using the following equation:

$$ \%\mathrm{original}\ \mathrm{wound}\ \mathrm{area}=\frac{\left(\mathrm{wound}\ \mathrm{area}\ \mathrm{at}\ \mathrm{day}\ \mathrm{X}\right)}{\left(\mathrm{wound}\ \mathrm{area}\ \mathrm{at}\ \mathrm{day}\ 3\right)}\times 100\% $$

#### Histological analysis of wound healing

Three mice were selected from each group, and the ulcer site and the surrounding tissue (approximately 1-cm radius) were excised, fixed in 4% paraformaldehyde for 24 h, and then transferred to PBS for storage at 4 °C until further use. Samples were embedded in paraffin, cut into three 5-μm-thick sections, and stained with hematoxylin and eosin (H&E). Five random visual fields of each section were selected to conduct histological analysis.

#### Reverse transcription (RT-PCR) and quantitative real time-polymerase chain reaction (qRT-PCR)

Total RNA was isolated using Trizol reagent (#RP1001, BioTeke, Beijing, China) and reverse-transcribed into cDNA using the First-Strand cDNA Synthesis System kit with random oligo primers. Samples were stored at − 20 °C until use in qRT-PCR. Primers were designed to be specific for mouse mRNA expression and for use in qRT-PCR analysis (Exicycler 96, Bioneer, South Korea). Nuclease-free water was used in place of the sample for negative control. β-actin was used as the internal control. Every sample was tested in duplicate. Melting curves were analyzed for each run to assess the presence of non-specific PCR products. The results were analyzed using Step One Software V2.1. The mRNA expression of interleukin-6 (IL-6), interleukin-10 (IL-10), TNF-α and transforming growth factor-β (TGF-β_1_) genes was calculated relative to the expression of the β-actin and according to the ^ΔΔ^Ct method.

#### Western blotting

Total protein was extracted using a standard procedure [30], separated on an 8–12% polyacrylamide gel (SDS-PAGE) and then transferred to a PVDF membrane (No. IPVH00010, Millipore, USA). vascular endothelial growth factor-A (VEGF-A, 1:500, No. WL03335, Wanleibio, Beijing, China), hypoxia inducible factor-1α (HIF-1α, 1:500, No. WL01607, Wanleibio, Beijing, China), transforming growth factor-β_1_ (TGF-β_1_; 1:500, No. WL02998, Wanleibio, Beijing, China) and β-actin (1:100, No. WL01372, Wanleibio, Beijing, China) were used as primary antibodies. Secondary antibodies were used at a 1:10,000 dilution, and protein expression was detected using an ECL assay (No. WLA003, Wanleibio, Beijing, China). Densitometry was conducted using the Gel Image Processing System (Gel-Pro Analyzer, Meyer Instruments, Houston, TX, USA). The immunoblots shown are representative of at least three independent experiments.

### Statistical analysis

Data are presented as the mean ± standard deviation (SD), and analyzed using two-way ANOVA followed by Tukey’s test for pairwise comparison using GraphPad Prism 7.00 (v7.Ii.0; San Diego, CA, USA). *P <* 0.05 was considered statistically significant.

## Results

### Preparation and characterization of LL-37/CS

**Fig. 1:**
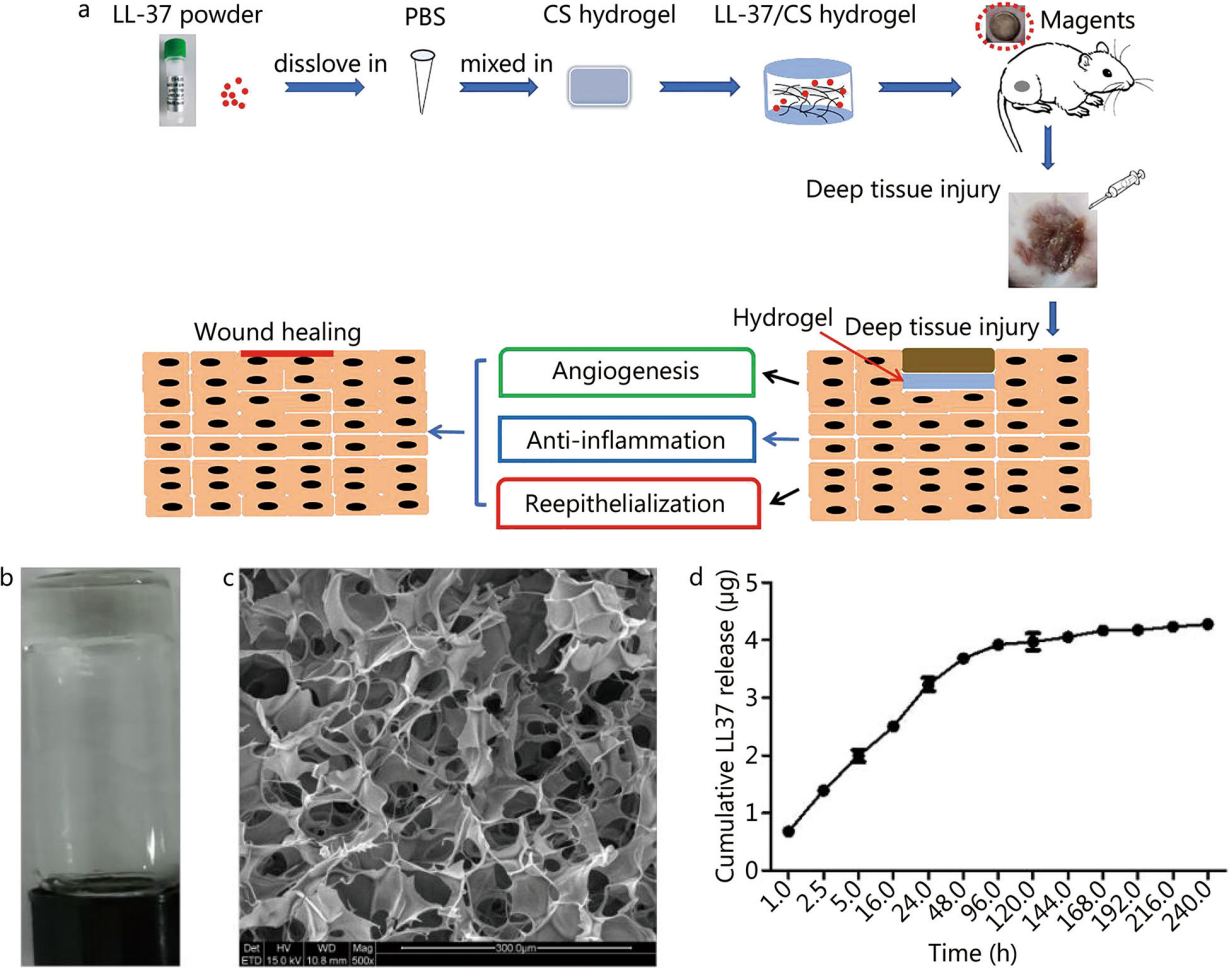
Schematic representation of mechanisms and Characterization of LL-37/CS hydrogel. a. This figure shows the mechanisms of action of LL37/CS hydrogel in deep tissue pressure injury healing processes; b. A representative image of the LL-37/CS hydrogel; *C. SEM* photographs of the LL-37/CS hydrogel. The scale bar indicates 500 nm; d. In vitro release of LL37 from the LL-37/CS hydrogel (plotted as μg cumulative release vs. time) (mean ± SD, *n* = 3)

CS hydrogels (2.5% w/v) containing various final concentrations of LL-37 were prepared: 0.1% w/w, 0.25% w/w and 0.5% w/w. Following gelation, stable hydrogels formed within minutes (Fig. 1a). Scanning electron microscopy (SEM) showed morphological characteristics common to freeze-dried hydrogels (Fig. 1b), indicating that all hydrogels had a porous network structure with homogeneous interconnectivity. The diameter of the porous network structure (range: 50–100 μm) was sufficient to incorporate and release LL-37 peptide. Encapsulation of LL-37 did not appear to affect the gelation performance of CS hydrogels. The encapsulation efficiency of LL-37 within CS hydrogels was 86.16% ± 2.61% (Fig. 1c) when LL-37 was loaded at 5 μg per mg CS hydrogel. Total amount of LL-37 within each hydrogel (0.1 ml) was 4.31 ± 0.13 μg.

### Cytotoxicity, immunoregulation and antimicrobial properties

**Fig. 2:**
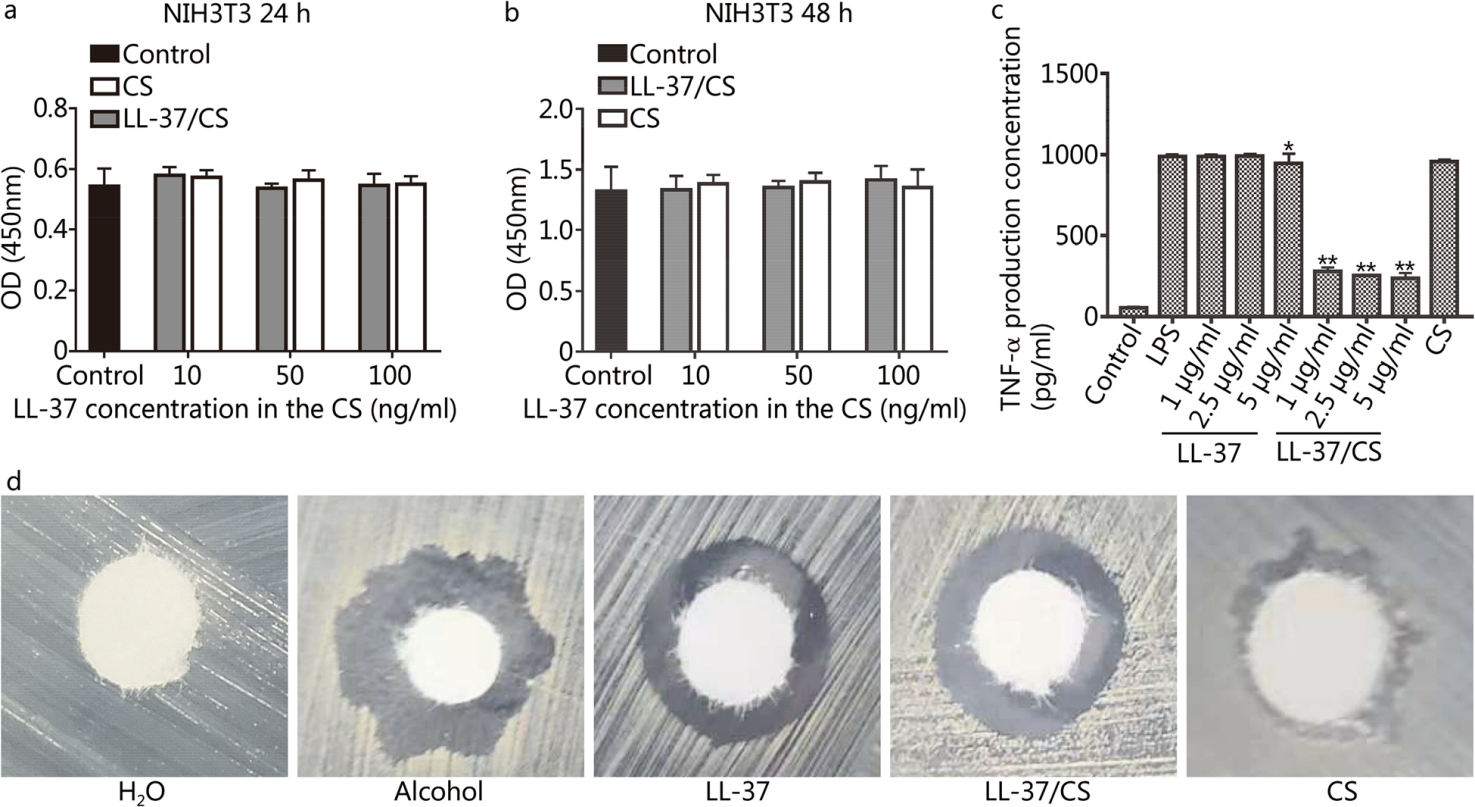
Activity of LL-37/CS in vitro. a-b. NIH3T3 cell viability was evaluated by the CCK-8 assay for 24 h and 48 h. NIH3T3 cells were treated with CS, LL-37 and LL-37/CS or PBS (control) (*n* = 6). There was no significant difference between the groups. c. Inhibition of TNF-α expression in RAW264.7 cells. Controls are cells without any addition. d. The antimicrobial results are given as the inhibition zones of bacteria relative to the control among the five groups. *P < 0.05 and **P < 0.01 compared with LPS group

Incubation of the NIH3T3 cells with either LL-37 or LL-37/CS hydrogels for 48 h did not decrease cell count (Fig. 2a-b). LPS-induced TNF-α release from macrophages was decreased by free LL-37 peptide as well as LL-37/CS hydrogel, with maximum activity at 5 μg/ml in hydrogel (Fig. 2c). Treatment with CS hydrogel alone did not affect TNF-α release. LL-37 peptide (5 μg/ml) and LL-37/CS hydrogel (containing 5 μg/ml LL-37) decreased the number of *Staphylococcus aureus* colony growth by approximately 40 and 25%, respectively (Fig. 2d). CS hydrogel treatment alone showed no significant antimicrobial activity.

### LL-37/CS hydrogels accelerate deep tissue injury wound healing

**Fig. 3:**
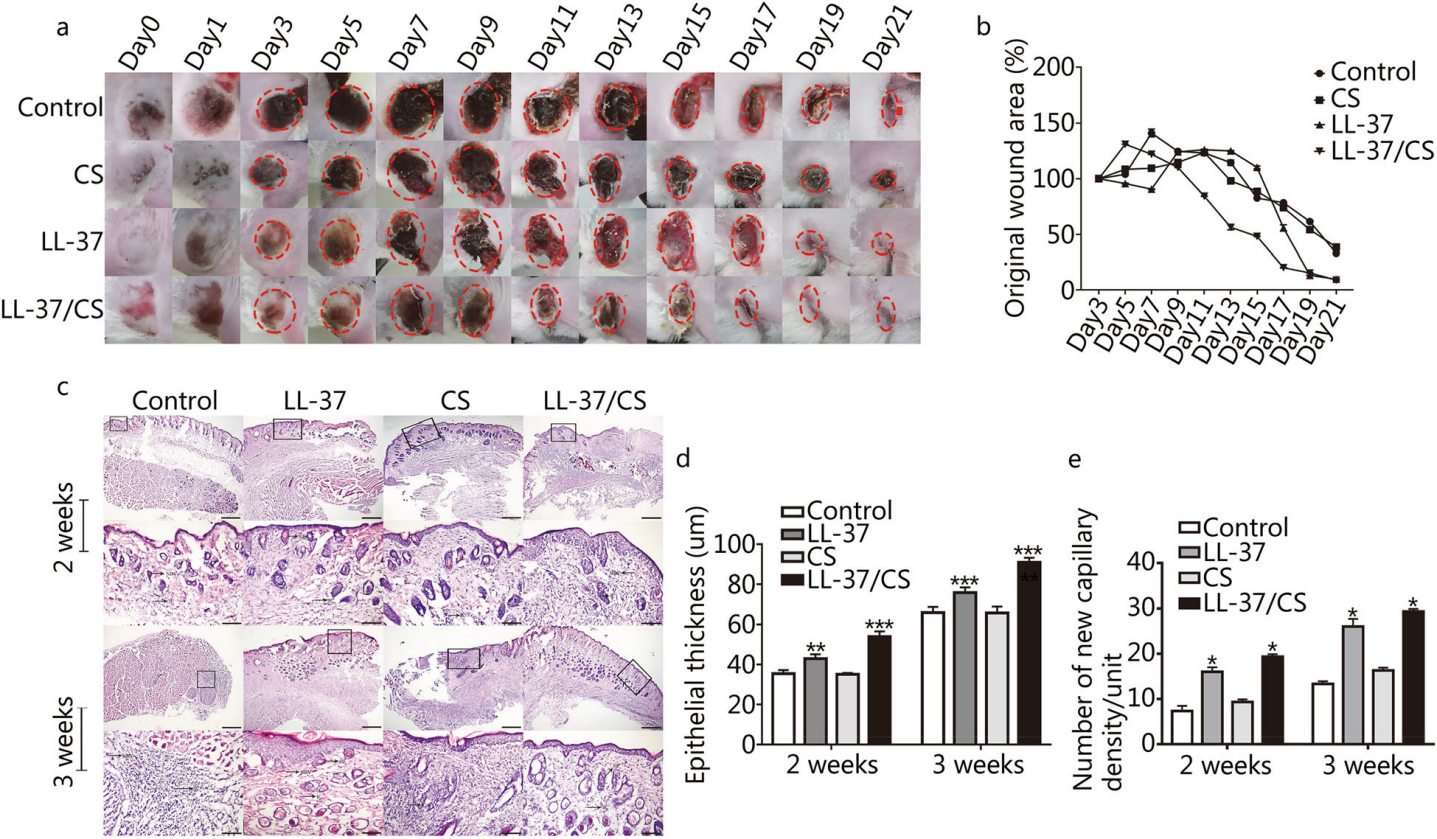
LL-37/CS accelerates wound healing. a. Representative images of wounds of four tested groups, ruler units in mm. b. The results are expressed as the percentage of the initial wound on days 3, 5, 7, 9, 11, 13, 15, 19, and 21. Statistical significance compared with control group (mean ± SD; *n* = 3). c. LL-37/CS facilitated an increase in the density of new capillaries and re-epithelialization of deep tissue injury. Wound sections (*n* = 3) were stained with hematoxylin and eosin (H&E). Representative sections are presented for all four groups on days 14 and 21 after application of treatment (× 10 and × 20, mean ± SD; *n* = 3). *P < 0.05, **P < 0.01 and ***P < 0.001 compared with control group

The largest wound closure was identified in the mice that received treatment with LL-37/CS hydrogels (Fig. 3a-b). The wound areas of control, CS, LL-37 and LL-37/CS groups were reduced to 33.05% ± 0.59, 38.52% ± 0.53, 79.78% ± 0.13 and 8.96% ± 0.58% at 21 days, respectively. The wound closure by LL-37/CS hydrogels was significant different in comparison to all other treatment groups for the vast majority of time points. Notably, the LL-37/CS group showed a wound area rate of 84.24% ± 0.25, 56.22% ± 3.91, 48.12% ± 0.28 and 20.14% ± 0.21% after 11, 13, 15 and 17 days, respectively, significantly higher than that of the other groups. On days 15 and 21, mice treated with LL-37/CS hydrogel displayed characteristics of rapid and effective wound healing in comparison to the control group. It was clear that in the LL-37/CS hydrogel treatment group, the epidermal and subepidermal layers were well defined and organized (Fig. 3c-e). At 2 weeks, there were fewer inflammatory cells and the new epidermis was formed to a greater extent in the LL-37 group and LL-37/CS group. The epithelial thickness in the control, LL-37, CS and LL-37/CS groups were 35.43 ± 1.80, 42.90 ± 2.25, 35.03 ± 0.76 and 53.98 ± 2.61 μm, respectively. The numbers of blood capillaries per field in the same four groups were 7.33 ± 1.15, 16 ± 1, 9.33 ± 0.58 and 19.33 ± 0.58, respectively. At 3 weeks, the wound surfaces treated by LL-37/CS were much smoother and the new tissue was more full-grown. The numbers of blood capillaries per field in the control, LL-37, CS and LL-37/CS groups were 13.33 ± 0.58, 26 ± 1.73, 16.33 ± 0.58 and 29.33 ± 0.58, respectively. The wound regions in the LL-37 and LL-37/CS groups had significantly more blood capillaries than that of control group (*P <* 0.01).

### LL-37/CS hydrogels upregulate VEGF-A, TGF-β_1_ and HIF-1α and downregulate TNF-α and IL-6 expression

**Fig. 4:**
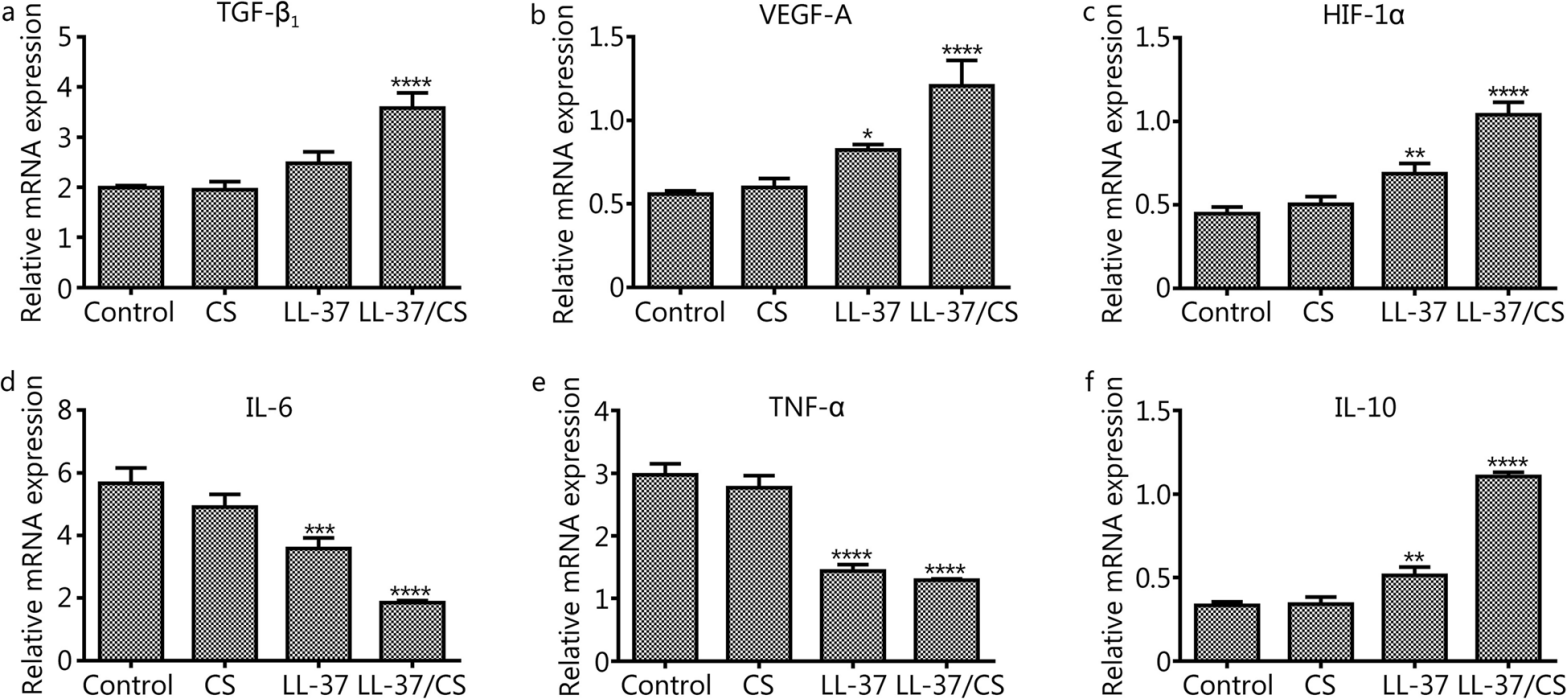
In vivo quantitative determination of mRNA expression in wound sites. a-f. TGF-β_1_, VEGF-A, HIF-1α, IL-6, TNF-α, and IL-10 expression at the mRNA level in deep tissue injury (mean ± SD; *n* = 3). The error bars indicate the standard deviation of the mean. ^*^P **<** 0.05, ** *P* **<** 0.01, ***P < 0.001 and ****P < 0.0001 compared with control group.

qRT-PCR revealed significantly higher expression of TGF-β_1_, VEGF-A and HIF-1α in the LL-37/CS hydrogel-treated group than in the control group on day 14 (Fig. 4a-c, *P <* 0.0001). However, there was no detectable difference in the mRNA expression of these genes between the untreated and CS hydrogel-treated group. Higher VEGF-A and HIF-1α expression was also observed in mice receiving free LL-37 in comparison with the control. IL-6 and TNF-α expression was lower on days 14 in the LL-37/CS hydrogel and LL-37 peptide-treated groups than that in control group (Fig. 4d-e, *P <* 0.001). No significant differences were observed between control and CS hydrogel-treated groups. IL-10 was increased in the LL-37/CS hydrogel-treated and LL-37 peptide-treated groups compared with control group (Fig. 4f, *P <* 0.01).

### LL-37/CS hydrogel upregulates HIF-1α, TGF-β and VEGF-A expression

**Fig. 5:**
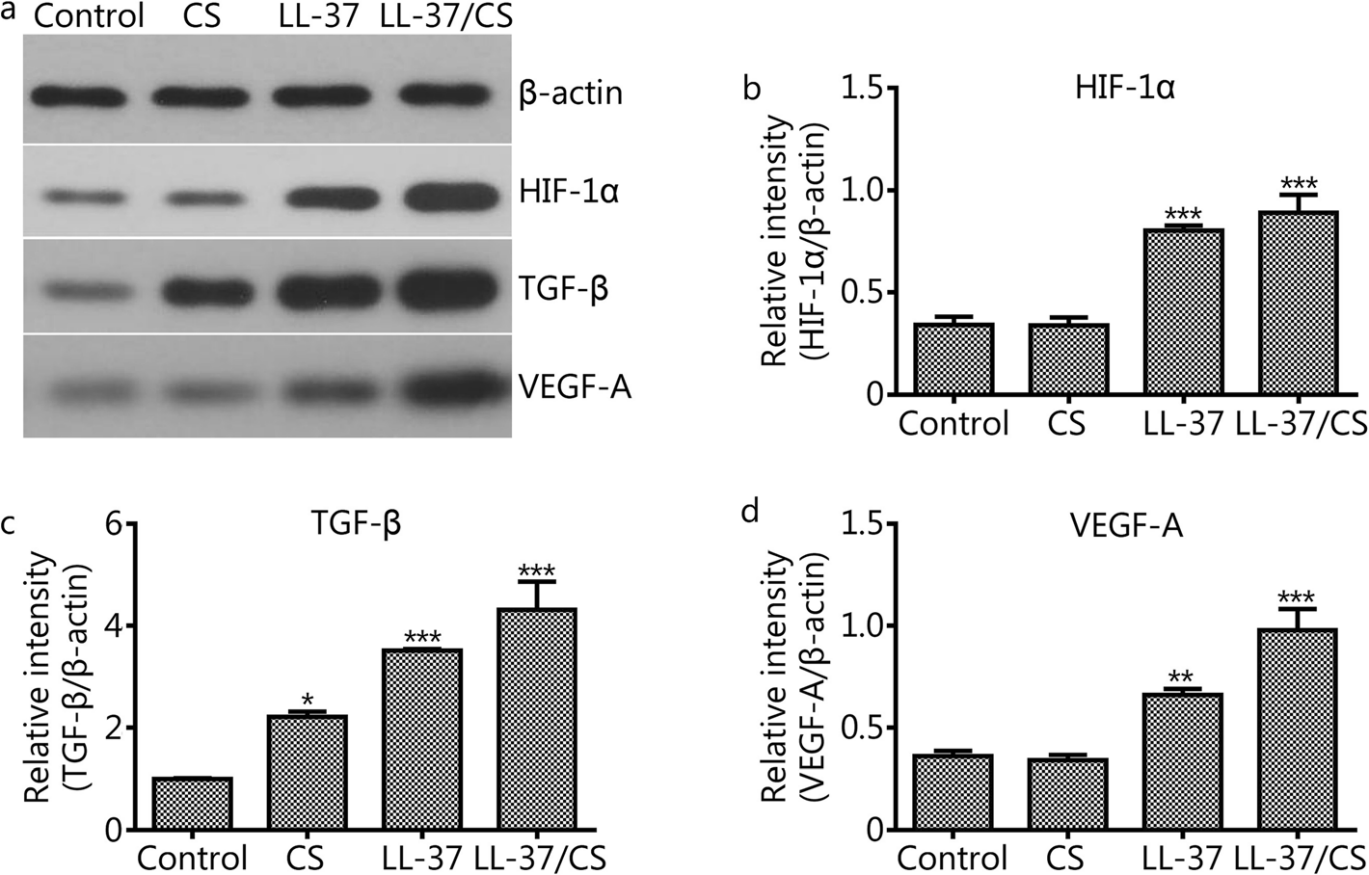
Results of Western blotting. This figure shows the changes in the protein expression of HIF-1α, TGF-β and VEGF-A in wound tissue; β-actin was used as a loading control. a-d. The relative protein expression of HIF-1α, TGF-β_1_ and VEGF-A was calculated as integrated density values. The error bars indicate the standard deviation of the mean. *P < 0.05, **P < 0.01 and ***P < 0.001 compared with control group.

Western blotting showed that the relative intensity of HIF-1α in control, CS, LL-37 and LL-37/CS groups were 0.36 ± 0.03, 0.34 ± 0.03, 0.66 ± 0.03 and 0.98 ± 0.1, repectively (Fig. 5b). The relative intensity of TGF-β in the same four groups were 1 ± 0.02, 2.22 ± 0.1, 3.52 ± 0.03 and 4.32 ± 0.56, respectively (Fig. 5c). And the relative intensity of VEGF-A in the same four groups were 0.36 ± 0.03, 0.34 ± 0.03, 0.66 ± 0.03 and 0.978 ± 0.1, repectively (Fig. 5d). Notely, LL-37/CS hydrogel and LL-37 peptide treatments increased HIF-1α, TGF-β and VEGF-A proteins in comparison to the control group (*P <* 0.01).

## Discussion

In this study, LL-37/CS hydrogels were injected subcutaneously into the wound site of deep tissue injuries. LL-37/CS hydrogels had an increased capacity to drive wound closure and to improve re-epithelialization by keratinocytes. In addition, the formulation and generation of LL-37/CS hydrogel used in the present study displayed excellent cytocompatibility and no cytotoxic effects in vitro (Fig. 2).

The pathological changes relating to symptoms of deep tissue injuries and pressure wounds were evident in control group and were observed to be resolved following treatment with LL-37/CS hydrogels (Fig. 3). Previous research has shown that ischemia-reperfusion injury can promote and aggravate deep tissue injuries and even result in limb gangrene or shedding [5, 31], suggesting that tissue hypoxia may play a prominent role in driving the complications associated with deep tissue injuries. Anti-inflammation and angiogenesis mechanisms were shown to be important factors in limiting damage to deep tissue muscle injuries [32, 33]; thus, targeting these fundamental mechanisms of wound healing may help to limit damage resulting from pressure wounds. TNF-α is the initiator of inflammatory responses in ischemia-reperfusion injury, resulting in the enhancement of antibody-dependent cell-mediated cytotoxicity, stimulation of cell degranulation and secretion of myeloperoxidases [34, 35]. IL-6 activates B-cells following its secretion by macrophages, lymphocytes and epithelial cells. IL-6 has also been shown to have dual functionality, either causing or inhibiting inflammation, depending on the chronicity of tissue trauma [36, 37]. In this study, tissue mRNA expression of the inflammatory factors IL-6 and TNF-α was determined by qRT-PCR, as shown in Fig. 4. In previous studies, it was found that LL-37 exerts a protective effect against inflammatory damage by inhibiting the activation of certain enzymes or activators of inflammatory factors [14, 38, 39], observations that are in keeping with our findings.

New blood vessel formation is also critical for tissue repair, as the provision of oxygen and nutrients to the wound site facilitates tissue regeneration [40]. VEGF is an endothelial-specific angiogenic factor that promotes endothelial cell proliferation, migration, and lumen formation and increases vascular permeability [41]. In this study, we demonstrated that LL-37 accelerated wound healing rates and stimulated the production of VEGF-A within wound tissue (Fig. 4). These results are in keeping with the findings of previous studies, which also demonstrated that LL-37 induced VEGF-A in human keratinocytes.

In this study, we show that the mechanism of LL-37 induction of VEGF-A production is regulated by HIF-1α. HIF-1α is an important nuclear transcriptional regulator involved in cellular adaptation to hypoxia and has roles in regulating hypoxic tissue angiogenesis, cell proliferation and cell survival [42]. Previous studies reported that HIF-1α upregulated VEGF expression and promoted angiogenesis, which suggests that it is vital in the rescue of tissue repair following ischemia-reperfusion-associated injuries. In this study, it was found that the expression of VEGF in the treated group was significantly higher than that in control group at day 14 after wounding. The data showed that HIF-1α had a positive correlation with VEGF protein expression, suggesting that HIF-1α is closely related to angiogenesis in deep tissue injuries (Figs. 4 and 5). TGF-β is a multifunctional cytokine that promotes angiogenesis after ischemia-reperfusion [43]. In our current work, the expression of TGF-β_1_ was detected in the different treatment groups by qRT-PCR on day 14 of wound healing. The results indicated that the mRNA expression of TGF-β within deep tissue injuries significantly increased after LL-37/CS hydrogel treatment. Therefore, LL-37/CS hydrogels may convey therapeutic action through the upregulation of pro-angiogenic growth factor expression, subsequently increasing blood supply while promoting the generation of granulation tissue and protecting against tissue matrix catabolism.

The local treatment of deep tissue injuries with LL-37/CS hydrogels shown in this investigation offers a promising therapy for deep tissue and pressure-associated injuries. It was previously suggested that LL-37 is involved in angiogenesis. Indeed, LL-37 has been shown to be associated with wound healing in chronic wounds, namely, diabetic foot ulcers. Our results showed that LL-37 hydrogels directly increased angiogenesis and pro-healing cytokine production in deep tissue injuries, suggesting that the outcomes from LL-37/CS hydrogel treatment in deep tissue injury healing may also be applicable to chronic nonhealing wounds or broader ischemic tissue injuries.

## Conclusion

LL-37-loaded CS hydrogels were successfully fabricated and could improve wound healing in deep tissue pressure injuries. Our results showed that topically injected LL-37/CS hydrogels could enhance anti-inflammatory and pro-angiogenic cytokine production in wounds of deep tissue injuries, overcoming the chronic inflammation and poor microcirculation issues commonly observed in chronic wound environments. The LL-37/CS hydrogel could effectively deliver LL-37 peptide to the wound site and produce antimicrobial and pro-healing activity.

## Data Availability

The datasets during and/or analyzed during the current study are available from the corresponding author on reasonable request.

## Abbreviations

ANOVA: Analysis of variance
CS: Chitosan
CCK-8: Cell Counting Kit-8
ELISA: Enzyme-linked Immunosorbent Assay
FBS: Fetal bovine serum
H&E: Hematoxylin and eosin
HIF-1α: Hypoxia-inducible factor-1α
IL-6: Interleukin-6
IL-10: Interleukin-10
LL-37: LL-37 peptide
LL-37/CS hydrogel: LL-37 loaded chitosan hydrogels
LPS: Lipopolysaccharide
PBS: Phosphate-buffered saline
qRT-PCR: Quantitative real-time polymerase chain reaction
RT-PCR: Reverse transcription polymerase chain reaction
SEM: Scanning electron microscope
SD: Standard deviation
TLR: Toll-like receptors
TNF-α: Tumor necrosis factor-α
TGF-β_1_: Transforming growth factor-β_1_
VEGF-A: Vascular endothelial growth factor-A
VLUs: Venous leg ulcers

## References

[1] Artico M, Dante A, D'Angelo D, Lamarca L, Mastroianni C, Petitti T (2018). Prevalence, incidence and associated factors of pressure ulcers in home palliative care patients: a retrospective chart review. Palliat Med.

[2] Demarré L, van Lancker A, van Hecke A, Verhaeghe S, Grypdonck M, Lemey J (2015). The cost of prevention and treatment of pressure ulcers: a systematic review. Int J Nurs Stud.

[3] Coyer F, Miles S, Gosley S, Fulbrook P, Sketcher-Baker K, Cook JL (2017). Pressure injury prevalence in intensive care versus non-intensive care patients: a state-wide comparison. Aust Crit Care.

[4] Zhao R, Liang H, Clarke E, Jackson C, Xue M (2016). Inflammation in chronic wounds. Int J Mol Sci.

[5] Peart J (2016). The aetiology of deep tissue injury: a literature review. Br J Nurs.

[6] Atkinson RA, Cullum NA (2018). Interventions for pressure ulcers: a summary of evidence for prevention and treatment. Spinal Cord.

[7] Espejo E, Andrés M, Borrallo RM, Padilla E, Garcia-Restoy E, Bella F (2018). Bacteremia associated with pressure ulcers: a prospective cohort study. Eur J Clin Microbiol Infect Dis.

[8] Lowry D, Saeed M, Narendran P, Tiwari A (2017). The difference between the healing and the nonhealing diabetic foot ulcer: a review of the role of the microcirculation. J Diabetes Sci Technol.

[9] Xhindoli D, Pacor S, Benincasa M, Scocchi M, Gennaro R, Tossi A (2016). The human cathelicidin LL-37--a pore-forming antibacterial peptide and host-cell modulator. Biochim Biophys Acta.

[10] Fumakia M, Ho EA (2016). Nanoparticles encapsulated with LL37 and serpin A1 promotes wound healing and synergistically enhances antibacterial activity. Mol Pharm.

[11] Agier J, Brzezińska-Błaszczyk E, Żelechowska P, Wiktorska M, Pietrzak J, Różalska S (2018). Cathelicidin LL-37 affects surface and intracellular toll-like receptor expression in tissue mast cells. J Immunol Res.

[12] Adase CA, Borkowski AW, Zhang LJ, Williams MR, Sato E, Sanford JA (2016). Non-coding double-stranded RNA and antimicrobial peptide LL-37 induce growth factor expression from keratinocytes and endothelial cells. J Biol Chem.

[13] Hou S, Sun X, Dong X, Lin H, Tang L, Xue M (2019). Chlamydial plasmid-encoded virulence factor Pgp3 interacts with human cathelicidin peptide LL-37 to modulate immune response. Microbes Infect.

[14] Steinstraesser L, Lam MC, Jacobsen F, Porporato PE, Chereddy KK, Becerikli M (2014). Skin electroporation of a plasmid encoding hCAP-18/LL-37 host defense peptide promotes wound healing. Mol Ther.

[15] Grönberg A, Mahlapuu M, Ståhle M, Whately-Smith C, Rollman O (2014). Treatment with LL-37 is safe and effective in enhancing healing of hard-to-heal venous leg ulcers: a randomized, placebo-controlled clinical trial. Wound Repair Regen.

[16] Garcia-Orue I, Gainza G, Girbau C, Alonso R, Aguirre JJ, Pedraz JL (2016). LL37 loaded nanostructured lipid carriers (NLC): a new strategy for the topical treatment of chronic wounds. Eur J Pharm Biopharm.

[17] Boateng J, Catanzano O (2015). Advanced therapeutic dressings for effective wound healing--a review. J Pharm Sci.

[18] Desfrançois C, Auzély R, Texier I. Lipid nanoparticles and their hydrogel composites for drug delivery: a review. Pharmaceuticals (Basel). 2018;11(4):E118. 10.3390/ph11040118 PMC6315535 30388738

[19] Das S, Baker AB (2016). Biomaterials and nanotherapeutics for enhancing skin wound healing. Front Bioeng Biotechnol.

[20] Naseri-Nosar M, Ziora ZM (2018). Wound dressings from naturally-occurring polymers: a review on homopolysaccharide-based composites. Carbohydr Polym.

[21] Gao J, Zheng W, Zhang J, Guan D, Yang Z, Kong D (2013). Enzyme-controllable delivery of nitric oxide from a molecular hydrogel. Chem Commun (Camb).

[22] Cong Z, Shi Y, Wang Y, Wang Y, Niu J, Chen N (2018). A novel controlled drug delivery system based on alginate hydrogel/chitosan micelle composites. Int J Biol Macromol.

[23] Frade ML, de Annunzio SR, Calixto GMF, Victorelli FD, Chorilli M, Fontana CR. Assessment of chitosan-based hydrogel and photodynamic inactivation against *Propionibacterium acnes*. Molecules. 2018;23(2):E473. 10.3390/molecules23020473 PMC6017752 29470387

[24] Xu J, Tam M, Samaei S, Lerouge S, Barralet J, Stevenson MM (2017). Mucoadhesive chitosan hydrogels as rectal drug delivery vessels to treat ulcerative colitis. Acta Biomater.

[25] Karri VV, Kuppusamy G, Talluri SV, Mannemala SS, Kollipara R, Wadhwani AD (2016). Curcumin loaded chitosan nanoparticles impregnated into collagen-alginate scaffolds for diabetic wound healing. Int J Biol Macromol.

[26] Vignesh S, Sivashanmugam A, Annapoorna M, Janarthanan R, Subramania I, Nair Shantikumar V (2018). Injectable deferoxamine nanoparticles loaded chitosan-hyaluronic acid coacervate hydrogel for therapeutic angiogenesis. Colloids Surf B Biointerfaces.

[27] Chen X, Cao X, Jiang H, Che X, Xu X, Ma B, et al. SIKVAV-modified chitosan hydrogel as a skin substitutes for wound closure in mice. Molecules. 2018;23(10):E2611. 10.3390/molecules23102611 PMC6222830 30314388

[28] Furuike T, Komoto D, Hashimoto H, Tamura H (2017). Preparation of chitosan hydrogel and its solubility in organic acids. Int J Biol Macromol.

[29] Sun Y, Liu Y, Liu W, Lu C, Wang L (2015). Chitosan microparticles ionically cross-linked with poly(γ-glutamic acid) as antimicrobial peptides and nitric oxide delivery systems. Biochem Eng J.

[30] Gopal A, Kant V, Gopalakrishnan A, Tandan SK, Kumar D (2014). Chitosan-based copper nanocomposite accelerates healing in excision wound model in rats. Eur J Pharmacol.

[31] Cui FF, Pan YY, Xie HH, Wang XH, Shi HX, Xiao J (2016). Pressure combined with ischemia/reperfusion injury induces deep tissue injury via endoplasmic reticulum stress in a rat pressure ulcer model. Int J Mol Sci.

[32] Makrantonaki E, Wlaschek M, Scharffetter-Kochanek K (2017). Pathogenesis of wound healing disorders in the elderly. J Dtsch Dermatol Ges.

[33] Arpino JM, Nong Z, Li F, Yin H, Ghonaim N, Milkovich S (2017). Four-dimensional microvascular analysis reveals that regenerative angiogenesis in ischemic muscle produces a flawed microcirculation. Circ Res.

[34] Al Salihi MO, Kobayashi M, Tamari K, Miyamura T, Takeuchi K (2017). Tumor necrosis factor-alpha antagonist suppresses local inflammatory reaction and facilitates olfactory nerve recovery following injury. Auris Nasus Larynx.

[35] Hirosaki H, Maeda Y, Shimojima M, Maeda K, Iwata H, Takeyoshi M (2019). Effects of soluble tumor necrosis factor (TNF) on antibody-dependent cellular cytotoxicity of therapeutic anti-TNF-alpha antibody. Immunol Investig.

[36] Jordan SC, Choi J, Kim I, Wu G, Toyoda M, Shin B (2017). Interleukin-6, a cytokine critical to mediation of inflammation, autoimmunity and allograft rejection: therapeutic implications of IL-6 receptor blockade. Transplantation.

[37] Yang G, Gu M, Chen W, Liu W, Xiao Y, Wang H (2018). SPHK-2 promotes the particle-induced inflammation of RAW264.7 by maintaining consistent expression of TNF-α and IL-6. Inflammation.

[38] Yu X, Quan J, Long W, Chen H, Wang R, Guo J (2018). LL-37 inhibits LPS-induced inflammation and stimulates the osteogenic differentiation of BMSCs via P2X7 receptor and MAPK signaling pathway. Exp Cell Res.

[39] Sun L, Wang W, Xiao W, Yang H (2016). The roles of cathelicidin LL-37 in inflammatory bowel disease. Inflamm Bowel Dis.

[40] Zhao X, Liu HQ, Li J, Liu XL (2016). Endothelial progenitor cells promote tumor growth and progression by enhancing new vessel formation. Oncol Lett.

[41] Jia J, Ye T, Cui P, Hua Q, Zeng H, Zhao D (2016). AP-1 transcription factor mediates VEGF-induced endothelial cell migration and proliferation. Microvasc Res.

[42] Park K, Lee HE, Lee SH, Lee D, Lee T, Lee YM (2017). Molecular and functional evaluation of a novel HIF inhibitor, benzopyranyl 1,2,3-triazole compound. Oncotarget..

[43] Jarad M, Kuczynski EA, Morrison J, Viloria-Petit AM, Coomber BL (2017). Release of endothelial cell associated VEGFR2 during TGF-β modulated angiogenesis *in vitro*. BMC Cell Biol.

